# Prevalence and Case Fatality of Congenital Heart Disease in Pakistani Infants: A 11‐Year Retrospective Time‐Series Analysis

**DOI:** 10.1002/hsr2.72812

**Published:** 2026-07-20

**Authors:** Ijaz ul Haq, Muhammad Imran Khan, Amir Muhammad, Mehwish Fayaz, Majid Ali, Numan Khan, Ibrahim Bin Afsar Jan, Guo‐ying Huang, Hu Xiaojing

**Affiliations:** ^1^ Fujian Key Laboratory Of Neonatal Diseases, Children's Hospital of Fudan University at Xiamen, Xiamen Children's Hospital Xiamen Fujian China; ^2^ Nursing Department Children's Hospital of Fudan University Shanghai China; ^3^ Department of Public Health & Nutrition The University of Haripur Haripur Khyber Pakhtunkhwa Pakistan; ^4^ Pediatrics Department Lady Reading Hospital, MTI Peshawar Pakistan; ^5^ Saidu Teaching Hospital Saidu Sharif Khyber Pakhtunkhwa Pakistan; ^6^ Paediatrics & Neonates Department Hayatabad Medical Complex Peshawar Pakistan; ^7^ Shanghai Key Laboratory of Birth Defects Shanghai China

**Keywords:** case fatality rate, congenital heart disease, neonates, post‐neonatal infants, prevalence

## Abstract

**Background and Aims:**

Congenital heart disease (CHD) is a significant cause of infant mortality. In Pakistan, inadequate screening in healthcare settings and under‐reporting of CHD cases contribute to high mortality and morbidity rates. This study aimed to determine the prevalence and case fatality rate of CHD over a decade among neonates and post‐neonatal infants in Buner, Khyber Pakhtunkhwa (KP), Pakistan. Clarifying the epidemiological profile of CHD in this region is vital for strengthening early detection in newborns.

**Methods:**

This retrospective, single‐center study was conducted at the District Headquarter Hospital, District Buner. Data related to various parameters were extracted from hospital records and presented as prevalence and case fatality rate (CFR). Time‐series analysis was performed to assess trends over the years.

**Results:**

During the study period, a total of 733 cases of CHD were identified among 78,713 newborns. Of these cases, 415 (56.6%) were male infants, while 318 (43.4%) were female. The prevalence rate varied, with a peak in 2023 at 17.05 per 1000 and a trough in 2018 at 5.15 per 1000. Over the 11 years, the overall CFR was 36.7%, with the highest in 2021 at 68.1% and the lowest in both 2015 and 2018 at 27.7% each. The number of CHD cases increased significantly over time, rising from 47 (0.86%) in 2013 to 139 (1.7%) in 2023.

**Conclusion:**

The data indicate a consistent rise in CHD cases, along with an increasing case fatality rate and prevalence over the years in District Buner, Pakistan. This study underscores the need for improved screening protocols for CHD and the adoption of electronic health records to enable timely reporting of CHD cases among neonates and post‐neonatal infants.

## 1 Introduction

Congenital heart disease (CHD) is a group of structural or functional abnormalities in the heart present at birth [1] affecting the heart walls, valves, blood vessels, or overall cardiac structure [2]. Most cases are asymptomatic and are typically identified during routine neonatal examinations or later in life. Globally, it is the most common type of birth defect, accounting for approximately 1% of all live births. The prevalence is notably higher and more severe in males, with a rate of 9.5 per 1000 live births [3], representing nearly 25% of all congenital malformations [4].

The exact etiology of CHD remains unclear but is believed to involve multifactorial causes, including genetic and environmental factors. Genetic variations can lead to heterogeneous disorders of the heart muscle, characterized by structural and electrical abnormalities, such as pediatric cardiomyopathy and other electrical diseases, with a prevalence of 1.1–1.5 per 100,000 children under 18 years of age [5]. Common risk factors, including a family history of CHD, consanguinity, advanced maternal age, limited prenatal care, and pregnancies with inadequate nutrition and folic acid intake, as well as infections, smoking, radiation exposure, and medications taken during pregnancy, have significant implications [6]. Most cases are not curable and require continuous long‐term medical care. Clinical factors such as the severity of the cardiac lesion, prematurity, and comorbidities influence mortality; however, the impact of social drivers, including race and ethnicity, is often overlooked [7]. Early detection in newborns is critical for reducing disability and mortality rates. The death or disability of affected infants is often significantly linked to delayed diagnosis, missed cases, or treatment delays. Pulse Oximetry (POX) serves as a key indicator for identifying CHD. Based on this, Professor Guoying Huang's research team has developed a novel “dual‐index” screening method for early detection. This approach integrates POX with cardiac murmur auscultation to screen for CHD in newborns within 6 to 72 h after birth. It effectively identifies suspected cases in newborns and enables timely diagnosis [8, 9, 10]. In a developing country such as Pakistan, this approach could potentially be adapted in the future to establish a standardized screening protocol.

However, determining the prevalence of CHD among newborns in different countries and regions serves as a critical foundation for advancing further screening research. It is prevalent worldwide, affecting both the most and least developed regions. For instance, in the United States (US), the prevalence is 8–10 per 1000 live births. As the population grows at an annual rate of 4.5%, CHD is projected to remain the most common congenital defect, with more adults than children under 18 years of age affected. Denmark and Australia report significant rates, 6.1 and 7.65 per 1000 live births, respectively [11]. In Turkey, the reported prevalence was 27.8 per 10,000 live births in a cohort from 2018 to 2020 [12], while neural tube defects were reported at 27.5 per 10,000 live births [13]. In Asia, the prevalence is highest in China (22.9 per 1000), followed by India (19.4 per 1000), Iran (8.6 per 1000), and then Pakistan (3.4 per 1000). In Pakistan, the most commonly found types include atrial septal defect (ASD) at 40%, ventricular septal defect (VSD) at 35%, aortic stenosis (AS) at 10%, and atrio‐ventricular septal defect (AVSD) at 5% of the total cases [14]. It is important to note that home delivery services provided by traditional birth attendants are a common practice in most parts of Pakistan. In such cases, neonates are not usually screened, and the true prevalence may be higher. This is highlighted by Agadoorappa et al., who investigated ethnic variations by reviewing a large cohort of newborns at birth. They found that children with parents from Pakistan have a higher prevalence compared to those from other countries [15]. Similarly, Su et al. suggested that China, Pakistan, India, and Nigeria have the highest mortality rates and contribute approximately 39.7% to global deaths from CHD [16]. A recent study published in “The Lancet Child & Adolescent Health” reported an 18.7% increase in cases since 1990, with 12 million individuals currently living with the condition and a 34.5% decline in mortality [17], indicating advancements in management. Furthermore, more CHD‐related deaths occur in low‐ and middle‐income countries compared to high‐income countries, highlighting the importance of early screening and management in low‐income settings [17]. Over the past decade, the approach to managing CHD in Pakistan has evolved, encompassing diagnostic procedures, medical treatment, palliative care, corrective surgeries, and public awareness initiatives. In this modern era, despite the wealth of technology and expertise at our disposal, it remains one of the most fatal disorders and is the second‐highest cause of mortality in children under the age of 5 [18].

In Pakistan, male preferences, a common practice in resource‐limited areas, result in delayed or neglected care for females, leading to health inequalities. The inaccessibility of specialized treatment, which is available only in major cities of Pakistan and has very few pediatric cardiologists, combined with poverty and the high cost of surgical interventions, contributes to an increase in prevalence and negatively affects health outcomes [19]. Despite awareness of the burden that CHD places on healthcare systems, developing countries such as Pakistan experience higher mortality and morbidity rates due to limited diagnostic facilities for early detection [20]. This study aims to evaluate the prevalence and CFR of CHD among newborns, neonates, and post‐neonatal infants at the District Headquarter (DHQ) Hospital Daggar, Buner, over the past decade (2013–2023). By examining local trends and related mortality, the research seeks to contribute to enhancing healthcare policies and improving the management and care of CHD in Pakistan.

## 2 Methods

### 2.1 Study Setting and Design

A retrospective facility‐based study was conducted at the DHQ Daggar District of Buner, Pakistan. This is a secondary care facility with well‐established obstetric and labor services. Early nursing care and screening services for congenital defects are provided and records are maintained.

### 2.2 Study Population

The study population comprised neonates (0–28 days old) and post‐neonatal infants (up to 6 months) diagnosed with CHD during the study period from 2013 to 2023. We extracted data on infants who met the predefined inclusion criteria from secondary sources obtained from the hospital's nursery ward records. It included the age of the child at the time of diagnosis, gender, and information regarding his discharge, referral, or death. Similarly, the number of CHD cases and deliveries, mortality rate per 100 cases, were recorded and utilized to estimate the annual prevalence and case fatality rates among neonates and post‐neonatal infants.

### 2.3 Inclusion and Exclusion Criteria

Neonates (0–28 days old) and post‐neonatal infants (up to 1 year old) born in this hospital or referred and diagnosed via echocardiography were admitted to the DHQ Hospital in Daggar, Buner, from January 1, 2013, to December 31, 2023. Patients with complete medical records, including follow‐up data and demographic information, were included in the study for retrospective review.

**Figure 1:**
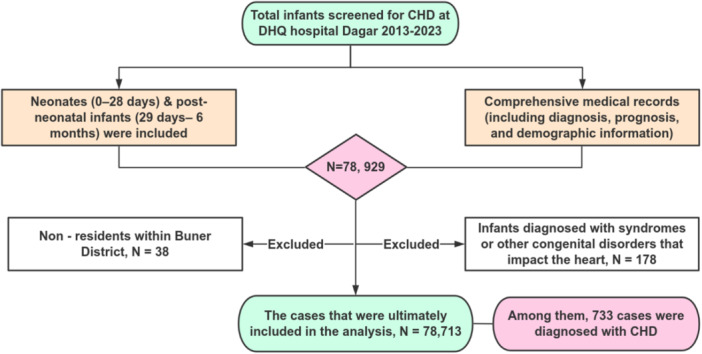
Flowchart illustrating the inclusion and exclusion criteria in relation to the total number of congenital heart disease (CHD) cases.

Patients with incomplete medical records missing critical information about their diagnosis, treatment, or outcomes, and those with complex genetic syndromes primarily caused by non‐CHD conditions were excluded from the study. The total number of deliveries was 78713, with 733 CHD cases included in the final analysis (Flow Chart Figure 1).

### 2.4 Case Fatality Rate

CFR is the proportion of people who die from a specified disorder among all the diagnosed individuals in a specific period. It is calculated by dividing the number of deaths from a disorder over a defined period of time by the number of diagnosed individuals; the obtained ratio is then multiplied by 100 to get a percentage, often used for the prognosis of a disorder [21].

### 2.5 Data Collection

The data collection method utilized secondary sources, where data from newborn patients already registered in the hospital's records were documented. The hospital lacked electronic data, so all information was obtained from paper registers. Trained researchers, skilled in extracting secondary data from non‐electronic sources, collected the data, adhering to study‐specific variables and ensuring credibility and reliability through cross‐checking. Before data collection, the researchers underwent training to extract secondary data from hospital records, which included understanding medical record numbers, demographic data, echocardiogram reports, and evaluating hospital admission and discharge slips. All infants born during the study period, who visited DHQ hospital Dagar, were included regardless of their admission status, such as newborns with minor defects requiring conservative management who were sent home and advised to follow up, or those referred to other facilities for specialized management.

### 2.6 CHD Diagnosis

Diagnostic procedures for CHD were conducted in accordance with guidelines established by the American Heart Association and the European Society of Cardiology [22]. Newborns were examined at the outpatient department following birth. Those exhibiting physical symptoms such as shortness of breath, cyanosis, persistent fatigue, impaired blood circulation, heart murmurs, and rapid breathing were referred for echocardiography. Moreover, children who had been discharged from the hospital but returned with these symptoms were also adviced to undergo echocardiography. Clinical checkups of the patients were performed by trained pediatricians or pediatric cardiologists in the hospitals. Echocardiography tests were conducted by specialized technicians and pediatric cardiologists.

### 2.7 Statistical Analysis

The general characteristics of the study population (neonates and post‐neonatal infants) are presented as frequency and proportion. The prevalence and CFR of CHD were calculated. Time series analysis was conducted to assess a year‐by‐year linear trend in prevalence or CFR from 2013 to 2023; the average annual percent change (AAPC) shows a summary statistic of the overall trend. We employed multiple linear regression analysis to identify risk factors associated with case fatality among infants diagnosed with CHD. In the univariate analysis, the associations between variables and case‐fatality rates were evaluated individually. Variables with a *p*‐value less than 0.20 were incorporated into the multivariate analysis. During the model‐fitting process, multicollinearity was examined using the Variance Inflation Factor (VIF). All variables in the model exhibited VIF values below 2, suggesting no significant collinearity issues, and were thus included in the final model. All statistical tests were two‐tailed, and significance was set at *p* < 0.05. Data was analyzed using the Statistical Package for the Social Sciences (SPSS version 26.0). Statistical analyses followed SAMPL guidelines. All analyses have been rechecked for accuracy.

## 3 Results

### 3.1 General Characteristics of Identified CHD Patients in District Headquarter Hospital Buner

Table S1 outlines the demographic and clinical characteristics of neonates and post‐neonatal infants diagnosed with CHD at the DHQ Hospital, Buner. Key variables include gender, age (in days), and clinical outcomes. Among the 733 patients, 415 (56.6%) were male and 318 (43.4%) were female. The age distribution indicates that the majority (65.8%) were between 1 and 28 days old, followed by 29–60 days (28.1%), and 61–89 days (6.1%). Various patient outcomes are detailed: 171 patients (23.3%) left against medical advice (LAMA), 164 (22.4%) were discharged, 160 (21.8%) were referred, and 247 (33.7%) died.

### 3.2 Year‐Wise Deliveries at District Headquarter Hospital Buner

Table 1 depicts the monthly delivery figures at DHQ Hospital, Buner, from 2013 to 2023. The data reveals fluctuations in both monthly and annual delivery numbers, yet indicates a steady increase in the overall deliveries over the years. For instance, total deliveries increased from 5481 in 2013 to a peak of 9091 in 2022, then decreased slightly to 8153 in 2023. The monthly delivery data also exhibits consistent seasonal patterns, with elevated delivery rates typically observed in months such as January, May, and December across several years.

**Table 1:** Year‐wise CHD cases and prevalence rate per 1000 population at District Headquarter Hospital Buner.

Year	Total number of deliveries	Number of CHD	Percentage (%)	Prevalence rate
2013	5481	47	0.9	8.6
2014	4565	44	1.0	9.6
2015	5529	39	0.7	7.1
2016	5924	48	0.8	8.1
2017	6693	52	0.8	7.8
2018	7958	41	0.5	5.2
2019	8319	43	0.5	5.2
2020	8265	62	0.7	7.5
2021	8735	95	1	10.9
2022	9091	123	1.3	13.5
2023	8153	139	1.7	17.0
Total	78,713	733	0.9	9.1

### 3.3 Yearly CHD Cases and Prevalence Rate per 1000 Population at District Headquarter Hospital Buner

Table 2 presents the annual number of cases and prevalence rate per 1000 population documented in association with the total number of deliveries at DHQ Hospital, Buner, spanning from 2013 to 2023.

**Table 2:** Prevalence rate per 1000 population at District Head Quarter Hospital Buner.

Year	Total number of deliveries	Number of CHD	Prevalence rate
2013	5481	47	8.6
2014	4565	44	9.6
2015	5529	39	7.1
2016	5924	48	8.1
2017	6693	52	7.8
2018	7958	41	5.2
2019	8319	43	5.2
2020	8265	62	7.5
2021	8735	95	10.9
2022	9091	123	13.5
2023	8153	139	17.0
Total			9.1

Over the years, there have been 78,713 deliveries, with 733 cases identified. Notably, the number of cases increased from 47 in 2013 to 139 in 2023. The year 2023 saw the highest number of cases, with 139 cases out of 8153 deliveries. Similarly, the prevalence rate undergoes annual fluctuations, reaching a peak of 17.05 per 1000 in 2023 and a trough of 5.15 per 1000 in 2018. The overall prevalence rate over the 11 years is 9.13 per 1000 deliveries. This persistent increase in the prevalence rate suggests an upward trajectory in the number of cases over time.

### 3.4 Case Fatality Rate and Mortality per 100 Population Over the Years at District Headquarter Hospital Buner

Table 3 summarizes the CFR and mortality rates among CHD patients from 2013 to 2023. The overall mortality rate over the 11‐year period is 0.31 per 100 population. Yearly mortality rates fluctuate, with the highest rate in 2023 (0.58) and the lowest in 2018 (0.16). The peak number of CHD‐related deaths occurred in 2023, with 47 deaths out of 139 cases, corresponding to a CFR of 33.8%. The data reveal a steady increase in the total number of cases, accompanied by a rising CFR within this age group. This indicates a significant burden of CFR associated with CHD in the hospital over time.

**Table 3:** Annual case fatality rate percentages and mortality rate per 100 population at the District Headquarter Hospital Buner.

Year	Number of CHD	Case fatality rate, CFR, *N* (%)	Mortality rate per 100
2013	47	15 (31.9)	0.27
2014	44	15 (31.9)	0.33
2015	39	13 (27.7)	0.24
2016	48	16 (34.0)	0.27
2017	52	18 (38.3)	0.27
2018	41	13 (27.7)	0.16
2019	43	15 (31.0)	0.18
2020	62	21 (44.7)	0.25
2021	95	32 (68.1)	0.37
2022	123	42 (34.1)	0.46
2023	139	47 (33.8)	0.58
Total	733	247 (33.7)	0.31

*Note:* Mortality rate is calculated as [(number of deaths/number of deliveries) × 100].

### 3.5 Average Annual Percent Change (AAPC) From 2013 to 2023

**Figure 2:**
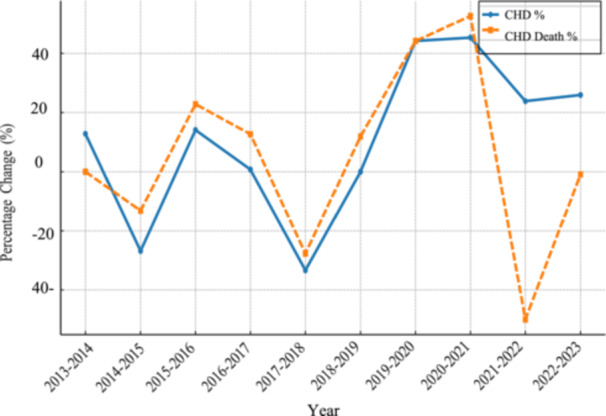
Graphical representation of average annual percentage change (AAPC) trends of CHD and mortality percentages (2013 − 2023). The 11‐year trend depicts the percentages of CHD and mortality from 2013 to 2023.

Table S2 and Figure 2 highlight the year‐on‐year changes in CHD percentages and death percentages at the DHQ Hospital, Buner, from 2013 to 2023. The prevalence exhibited a mix of increases and decreases over the years. Notable surges were observed between 2019 and 2020 (+44.23%) and 2020–2021 (+45.33%), indicating a significant rise in cases. Declines, such as from 2017 to 2018 (−33.33%), reflect a temporary decrease in identified cases.

Death percentages remained relatively stable during some periods, with slight fluctuations. However, notable increases, such as in 2020–2021 (+52.58%), indicate a rise in mortality among CHD cases. Conversely, sharp declines, like in 2021–2022 (−49.93%), show improvements in outcomes during certain years.

### 3.6 Associated Factors With Case Fatality of Congenital Heart Disease Among Neonates and Post‐Neonates

Table 4 shows the linear regression analysis identified several key factors associated with case fatality among infants. LAMA had a significantly higher risk of mortality (*p* < 0.001), highlighting the critical role of continuous medical care. Referral status was also a strong predictor of fatality (*p* < 0.001), suggesting that more severe cases were referred and carried a higher risk of death. Age was inversely associated with fatality (*p* = 0.041), indicating that younger infants, particularly those in the neonatal period, were more vulnerable. A significant upward trend in case fatality was observed over the years (*p* = 0.024), which may reflect either increasing severity.

**Table 4:** Factors associated with case fatality rates.

	Beta (unstandardized coefficient)	Standard error	Beta (standardized coefficient)	*p*‐value
Gender	0.024	0.018	0.054	0.17
Age	−0.002	0.001	−0.091	0.041
LAMA	0.183	0.026	0.312	< 0.001
Referral status	0.102	0.024	0.194	< 0.001
Years	0.007	0.003	0.145	0.024

Abbreviation: LAMA, left against medical advice.

## 4 Discussion

This study provides one of the first long‐term time series estimates of the prevalence and case fatality rate of CHD among neonates and post‐neonatal infants, using routinely collected clinical data from a resource‐constrained area. The findings of this retrospective study indicate an increasing prevalence and mortality at DHQ Hospital, Buner, over the last decade. A total of 733 cases were identified, with a prevalence rate of 9.13 per 1000 live births and a mortality rate of 36.7%, providing evidence to inform health system planning, early screening strategies, and the prioritization of pediatric cardiac care services. It also highlights important features that help understand the burden of CHD on the healthcare system, suggesting that addressing risk factors, gender disparities, and treatment gaps through a comprehensive strategy, including early screening and improving the accessibility and affordability of quality care, can be beneficial in reducing the prevalence and improving health outcomes in the local population.

The prevalence in our study was compared with that of a previous hospital‐based study in Karachi, Pakistan, which reported 3.4 per 1000. In contrast, China (22.9 per 1000) and India (19.4 per 1000) have a higher prevalence [14]. The reason might be the lack of national‐level data and previous studies on CHD in newborns in Pakistan. Therefore, there is a need for national‐level related studies. High prevalence could lead to high infant mortality. A Turkish study has reported an infant mortality rate of 8.8 per 10,000 live births specific to chronic CHD [23].

The findings indicate an upward trend in prevalence, rising from 8.5 per 1000 live births in 2013 to 17 in 2023. The highest prevalence, 17.05, was recorded in 2023. This increase can be attributed to heightened awareness and accessibility, advancements in screening, monitoring, and diagnostic practices, and infrastructure improvements. These findings are consistent with the global prevalence, which ranges from 8 to 12 per 1000 live births [16]. This study found a higher prevalence in males (56.6%), with similar findings reported by H. Mahmoud and S. Amin in 2023, suggesting that certain types of CHD, such as left heart syndrome and transposition of the great arteries, are more common in males [24]. Importantly, the majority (65.7%) of cases were diagnosed during the critical period, the first 20 days of life. However, a significant proportion (34.3%) were diagnosed after a delay of more than 20 days, indicating the progressive nature of the disease. This reinforces the need for early screening and monitoring, even before birth, to prevent missed early detection. A CFR above the global average (40%) aligns with the findings of a study conducted in Nigeria [25], which also reported low‐resourced environments with limited access to specialized cardiac care. The CFR fluctuated throughout the study period, peaking at 68.1% in 2021 and 44.6% in 2020, potentially due to the strain placed on healthcare systems by the COVID‐19 pandemic [26], which may have disrupted routine neonatal care in resource‐constrained areas such as Buner [27]. The lowest rate observed in 2015 and 2018 was 27.7%. The higher CFR can be attributed to the less developed healthcare and infrastructure at DHQ Hospital, Buner, as well as limitations in accessibility and availability of neonatal care and specialized pediatric cardiology services. Other possible causes of the higher CFR include the severity of heart problems, delays in referrals to specialized centers, and deficiencies in the neonatal critical care unit. As highlighted in previous studies, timely referrals and early cardiac care are critical factors in neonatal survival [27]. A high referral rate reflects an effective referral coordination mechanism; however, the elevated CFR suggests potential gaps in either the timeliness of transfers or the provision of critical care.

The most concerning finding of the study was the significant proportion (23.3%) of neonates who left against medical advice. This may contribute to an elevated CFR and is associated with multiple factors, including financial constraints [28], lack of knowledge among families, and the belief that treatment is ineffective. Among the remaining neonates, 29.2% were discharged and 22.1% were referred for specialized care, indicating a potential gap in specialized care services provided at this facility.

The total number of deliveries in the hospital during the study period was 78,713, with a significant increase observed in 2019. The data indicates an upward trend over the last 11 years (2013–2023), with the highest number of deliveries (9091) recorded in 2022 and the lowest (4565) in 2014. The number of CHD cases remained relatively stable in the early years of the study. The lowest number of cases [39] was documented in 2015, while the last 2 years demonstrated a significant rise, with 123 and 139 cases reported in 2022 and 2023, respectively. Similarly, the prevalence rate ranged from 5.15 in 2018 to 17 in 2023, reflecting a marked increase in the last 2 years, which may be attributed to improvements in early detection and diagnosis [29]. The data suggest that CHD and related deaths were not affected by fluctuations in delivery volume. However, maternal infections, diabetes, nutritional deficiency, and genetic predisposition may play a role in the prevalence and associated deaths [30].

In our study, a gender difference was also observed among neonates in the study area. Our findings were supported by a previous study, which stated that males have a higher risk of being born with severe CHD compared to females [31]. Another study reported a 2.6:1 male‐to‐female ratio among neonates [32]. A meta‐analysis also reported that the prevalence was higher among males than among females [33].

The data reflect fluctuating trends in CHD identification, prevalence, and outcomes over the decade, underscoring the need for continued monitoring, early diagnosis, and improved management. The DHQ Hospital in Buner lacks adequate facilities for early diagnosis and management. To address this, it is recommended to establish screening protocols and policies, invest in healthcare infrastructure, and train staff in early detection and management [34]. There is a need to enhance and optimize clinical records, particularly data pertaining to the demographic and socioeconomic circumstances, as well as essential medical records of mothers and children, to facilitate future research and planning. In this context, the birth statistics of the Turkish Statistical Institute (TSI) and the birth and death notification system can be adopted [23]. Furthermore, referral networks should be strengthened to ensure timely access to specialized cardiac care services [35]. A public health campaign should be launched to improve maternal health and prenatal care, focusing on mothers with risk factors and emphasizing the importance of early detection and continuity of care [36]. Collaboration with higher authorities and specialized cardiac care hospitals is essential to upgrade staff skills through training and experience‐sharing, particularly focusing on prenatal screening, dual‐index screening after birth, better‐equipped neonatal intensive care units, and expedited referral pathways to specialized cardiac centers. Future research should focus on identifying specific types of CHD in the region and their contributing factors, which could help reduce the high mortality rates associated with the defect.

## 5 Limitations

There was no complete data on the full addresses of CHD patients. Missing data in the registers is possible due to manual data entry, which increases the likelihood of bias and incomplete information. Record‐keeping limitations were evident, preventing us from stratifying the data into specific types of CHD. Although this study considered various aspects of CHD and related mortality, it was a retrospective study, which may have led to underreporting or inaccuracies in case documentation. The unavailability of data related to screening modification, such as the introduction of dual method or scaling up of pulse oximetry, could not evaluate the effects of evolving screening strategies on detection of cases and case fatality trends. As the hospital involved is a public facility in a remote district (Buner), primarily serving individuals from low socioeconomic backgrounds, patients from other socioeconomic groups may prefer private hospitals, potentially introducing selection bias. Conducted at a single hospital, the study may lack generalizability to the entire region. Nevertheless, this is the first study in the region to demonstrate an 11‐year trend in CHD.

## Conclusion

In conclusion, there has been a steady increase in CHD cases, along with a rising CFR and prevalence over the years in District Buner, Pakistan. This study highlights the critical need for capacity building in early detection and timely referral systems, improved screening mechanisms that utilize standard protocols such as POX across maternity and neonatal facilities, implementation of electronic health records, and the establishment of a national CHD registry to facilitate timely and accurate reporting among neonates and post‐neonatal infants. Furthermore, the health department should focus on enhancing healthcare facilities for addressing risk factors and managing infants to reduce prevalence and early deaths.

## Author Contributions

**Ijaz ul Haq:** conceptualization, methodology, writing – original draft. **Muhammad Imran Khan:** conceptualization, methodology, writing – original draft. **Amir Muhammad:** software, data curation. **Mehwish Fayaz:** investigation, validation. **Majid Ali:** investigation, validation. **Numan Khan:** formal analysis, data curation. **Ibrahim Bin Afsar Jan:** formal analysis, visualization. **Guo‐ying Huang:** project administration, resources, writing – review and editing, supervision. **Hu Xiaojing:** project administration, supervision, resources, writing – review and editing.

## Disclosure

All authors have read and approved the final version of the manuscript. Prof Xiaojing Hu had full access to all of the data in this study and takes complete responsibility for the integrity of the data and the accuracy of the data analysis.

## Ethics Statement

Ethical approval was obtained from the ethics committee of the Department of Public Health and Nutrition, The University of Haripur, Pakistan. All the patients have given their consent to use their data anonymously for research purposes.

## Conflicts of Interest

The authors declare no conflicts of interest.

## Transparency Statement

The lead author Prof Xiaojing Hu affirms that this manuscript is an honest, accurate, and transparent account of the study being reported; that no important aspects of the study have been omitted; and that any discrepancies from the study as planned (and, if relevant, registered) have been explained.

## Supporting information


Supporting File

Supporting File

## Data Availability

The datasets analyzed in the present study are archived within the institutional system of the first author. Access to these data may be requested by contacting the corresponding author via email, subject to approval based on legitimate research purposes and confidentiality requirements.
