# Supplementary material for: Prevalence and Case Fatality of Congenital Heart Disease in Pakistani Infants: A 11‐Year Retrospective Time‐Series Analysis
Source: Health Sci Rep. 2026 Jul 20;9(7):e72812. doi: 10.1002/hsr2.72812 (PMC13386109; doi:10.1002/hsr2.72812)
Supplement: Supplementary file 1 — Supporting File [file HSR2-9-e72812-s001.docx]

**Supplementary Table 1: General Characteristics of Identified CHD among Neonates and Post-neonatal Infants at District Headquarter Hospital Buner (n=733)**

| **Variables** | **Category** | **Frequency** | **Percentages（%）** |
| --- | --- | --- | --- |
| Gender | Male | 415 | 56.6 |
|  | Female | 318 | 43.4 |
| Age (Days) | 1-28 days | 482 | 66.5 |
|  | 28-60 days | 206 | 28.4 |
|  | 60-89 days | 45 | 6.2 |
| *Leave Against Medical Advice (LAMA) | Yes | 171 | 23.3 |
|  | No | 562 | 76.7 |
| Discharge | Yes | 164 | 22.4 |
|  | No | 569 | 77.6 |
| Referred | Yes | 160 | 21.8 |
|  | No | 573 | 78.2 |
| Death | Yes | 247 | 33.7 |
|  | No | 486 | 66.3 |

*Patients who leave the hospital and go home despite required treatment.
