# Supplementary material for: Prevalence and Case Fatality of Congenital Heart Disease in Pakistani Infants: A 11‐Year Retrospective Time‐Series Analysis
Source: Health Sci Rep. 2026 Jul 20;9(7):e72812. doi: 10.1002/hsr2.72812 (PMC13386109; doi:10.1002/hsr2.72812)
Supplement: Supplementary file 2 — Supporting File [file HSR2-9-e72812-s002.docx]

### ****Supplementary Table 2: Average Annual Percent Change (AAPC) from 2013 to 2023****

| **Year** | **CHD Percentage (%)** | **Death Percentage (%)** |
| --- | --- | --- |
| 2013-2014 | 12.79% | 0.00% |
| 2014-2015 | −26.80% | −13.17% |
| 2015-2016 | 14.08% | 22.82% |
| 2016-2017 | 0.70% | 12.65% |
| 2017-2018 | −33.33% | −27.68% |
| 2018-2019 | 0.00% | 11.91% |
| 2019-2020 | 44.23% | 44.19% |
| 2020-2021 | 45.33% | 52.58% |
| 2021-2022 | 23.85% | −49.93% |
| 2022-2023 | 25.93% | −0.88% |
